# Establishing F1A-CreER^T2^ Mice to Trace *Fgf1* Expression in Adult Mouse Cardiomyocytes

**DOI:** 10.3390/cells11010121

**Published:** 2021-12-30

**Authors:** Yi-Chao Hsu, Yu-Fen Chung, Mei-Shu Chen, Chi-Kuang Wang, Si-Tse Jiang, Ing-Ming Chiu

**Affiliations:** 1 Institute of Biomedical Sciences, Mackay Medical College, New Taipei City 252, Taiwan; hsuyc@mmc.edu.tw; 2 Department of Audiology and Speech Language Pathology, Mackay Medical College, New Taipei City 252, Taiwan; 3 Institute of Cellular and System Medicine, National Health Research Institutes, Miaoli 350, Taiwan; yufen@nhri.edu.tw (Y.-F.C.); meishu@nhri.edu.tw (M.-S.C.); 4 Department of Research and Development, National Laboratory Animal Center, National Applied Research Laboratories, Tainan 700, Taiwan; bujiii@nlac.narl.org.tw (C.-K.W.); stjiang@nlac.narl.org.tw (S.-T.J.); 5 Department of Life Sciences, National Chung Hsing University, Taichung 400, Taiwan; 6 Department of Internal Medicine, The Ohio State University, Columbus, OH 43210, USA

**Keywords:** FGF1, *Fgf1A* promoter, inducible Cre, cardiomyocyte

## Abstract

Fibroblast growth factor 1 (FGF1) regulates many biological and physiological processes. In mice, *Fgf1* gene contains at least three upstream promoters and are alternatively spliced to the first protein coding exon, giving rise to different *Fgf1* mRNA variants (1A, 1B and 1G). Among them, the *Fgf1A* transcript is predominantly expressed in the heart. FGF1 can induce cardiomyocyte regeneration and cardiogenesis in vitro and in vivo. Here, we generated a novel mouse line using the *Fgf1A* promoter (F1A) driving the expression of the inducible Cre recombinase (CreER^T2^). We firstly demonstrated that the highest mRNA expression of *CreER^T2^* were detected in the heart specifically of F1A-CreER^T2^ mice, similar to that of *Fgf1A* mRNA. The F1A-CreER^T2^ mice were crossed with ROSA26 mice, and the F1 mice were analyzed. The LacZ-positive signals were detected exclusively in the heart after tamoxifen administration. The CreER^T2^-mediated recombination in the tissues is monitored through LacZ-positive signals, indicating the in situ localization of F1A-positive cells. Consistently, these F1A-positive cells with RFP-positive signals or LacZ-positive blue signals were co-localized with cardiomyocytes expressing cardiac troponin T, suggesting cardiomyocyte-specific activation of *Fgf1A* promoter. Our data suggested that the F1A-CreER^T2^ mouse line could be used for time-dependent and lineage tracing of *Fgf1A*-expressing cells in vivo.

## 1. Introduction

The Cre/loxP site-specific recombination system has been widely used for the studies of promoter activation for genes of interest and conditional knockout mice [1]. The fusion of Cre recombinase with triple mutation including G400V/M543A/L544A of ligand binding domain of the human estrogen receptor (ER), CreER^T2^, showed responsive tamoxifen-inducibility [1,2]. To target and trace the lineage-specific cells in the cardiovascular system, Kimura et al. reported a ubiquitous CAG promoter or the cardiomyocyte-specific α-MHC promoter driven *HIF-1α*-ODD-CreER^T2^ transgene line. By crossing this line with ROSA26/tdTomato reporter line, they identified a population of cardiomyocytes with hypoxic characteristics, such as proliferation [3]. In addition, Cuervo et al. reported that *PDGFRβ*-P2A-CreER^T2^ mouse line can be used to target and trace NG2^+^, desmin^+^, PDGFRβ^+^ perivascular cells in the brain and retina when crossing with a fluorescent reporter mouse line. Furthermore, they demonstrated the feasibility to delete Notch signaling in pericytes surrounding the blood vessels, leading to the loss of smooth muscle cells in situ [4].

Fibroblast growth factor 1 (FGF1) is the prototype of FGF family proteins and is involved in a wide range of biological processes, especially in neural development and cardiovascular system through FGF1/FGF receptors (FGFRs) signaling [5]. There are four tissue-specific promoters 1A, 1B, 1C, 1D and their respective 5′-untranslated exons adjacent to each of the four promoters at the 5′-UTR of human *FGF1* gene to control the tissue-specific expression [6,7]. Similar to the human gene, the mouse *Fgf1* gene comprises at least three upstream promoters and their respective 5′-untranslated exons that are spliced to the first protein-coding exon, giving rise to *Fgf1* alternative transcripts (1A, 1B and 1G), each expressed in a tissue-specific manner [6,7]. The predominant expression of *Fgf1A* transcript is in the heart tissues; abundant expression of the *Fgf1B* transcript is detected in the brain and several glioblastoma cell lines [8,9,10]. *Fgf1G* transcript is expressed mostly in the liver and kidney. FGF1 plays important regulatory roles in cell survival [11], proliferation [12], regulation of neural stem/progenitor cells [13,14,15], learning and memory [16,17], metabolic homeostasis [18] and regulation of cardiogenesis [10,19,20,21].

In vitro, FGF1 has been shown to stimulate the neonatal cardiomyocytes cell cycle reentry through FGFR1/Fn14 pathway [21]. FGF1 has also been shown to be upregulated in the neonatal cardiomyocytes with *Cx43* knockdown [12]. FGF1 can regulate the cardiomyocyte differentiation from mouse embryonic stem cells through FGF1/FGFR/PKC signaling axis [20]. In vivo, FGF1 has been shown to induce cardiomyocyte regeneration and improve cardiac function after myocardial infarction [19]. Furthermore, FGF1 has been shown to prevent the diabetic cardiomyopathy through the reduction of oxidative stress [22,23]. Notably, FGF1/neurogenin1 can stimulate the proliferation of cardiomyocytes and facilitate the cardiac remodeling after myocardial infarction [24]. However, thus far, there is no genetic tool yet to trace the lineage of *Fgf1*-expressing cells in the heart. In this study, we report the generation of *Fgf1A*-CreER^T2^ transgenic mouse line and characterization of cell-specific expression of *Fgf1A* in heart. Our data showed that tamoxifen treatment of *Fgf1A* promoter (F1A)-CreER^T2^ mice showed Cre-mediated recombination in cardiomyocytes, while tamoxifen-induced recombination in other cell types was not observed. Considering the tissue-specific expression and the functional importance of *Fgf1A* in cardiogenesis in vitro and in vivo [19,20], a transgenic mouse line expressing tamoxifen-inducible CreER^T2^ driven by the endogenous *Fgf1A* promoter will be useful for in vivo time-dependent and lineage-tracing studies during heart development.

## 2. Materials and Methods

### 2.1. Generation of F1A-CreER^T2^ Transgenic Mice

**Figure 1:**
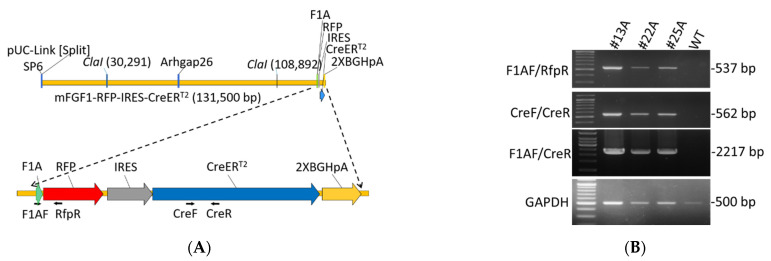
Construction map and genotyping of F1A-CreER^T2^ transgenic mice. (**A**) Construction map of F1A-CreER^T2^ transgenic mice. The transgene of F1A-RFP-IRES-CreER^T2^ was constructed using mouse *Fgf1A* promoter including *Fgf1A* promoter sequence comprising 127.7 kb of upstream sequences, followed by 714 bp of RFP gene sequences, 536 bp of IRES gene sequences, and 2001 bp CreER^T2^ sequences. Primers designed for genotyping: F1AF, RfpR, CreF and CreR. (**B**) Genotyping results of three F1A-CreER^T2^ founders: #13A, #22A and #25A. The amplicon of primer F1AF/RfpR is 537 bp; F1AF/CreR is 2,217 bp, and CreF/CreR is 562 bp.

A mouse bacterial artificial chromosome (BAC) clone RP23-193N23 including *Fgf1A* 5′UTR (127.7 kb), exon 1A, and 1B sequence was obtained from BACPAC Resources Center at the Children‘s Hospital of Oakland Research Institute, Oakland, CA, USA. The bicistronic expression cassette RFP-IRES-CreER^T2^–polyA was constructed by traditional cloning techniques, and was inserted into the *Fgf1A* exon 1A in the BAC clone by using the RED/ET recombination technique (Gene Bridges, Ltd., Heidelberg, Germany). Briefly, the 50-mer homologous arms (HR, red capital letters in Figure 1A) flanking the inserting site on the exon 1A was capped upon a counter selector rpsL-neo by polymerase chain reaction (PCR), and was electroporated into *E. coli* hosting the BAC clone to insert the rpsL-neo into exon 1A by homologous recombination. The RFP-IRES-CreER^T2^ –polyA cassette capping 50-mer homologous arms was then used to replace the counter selector in the exon 1A by homologous recombination so as to construct the transgene (Figure 1A). The detailed protocol was described in the instruction manual of the counter selection BAC modification kit (Gene Bridges, Ltd., Heidelber, Germany). The BAC transgene was purified, Not I digest, and pulsed-field gel electrophoresis to isolate the 131.5 kb transgene including the *Fgf1A* 5′UTR (127.7 kb) and expression cassette (3.8 kb) for C57BL/6 mouse pronuclear microinjection (Supplementary Figure S1). The F1A-CreER^T2^ transgenic mice were generated at the National Laboratory Animal Center in National Applied Research Laboratories. All mouse procedures performed were reviewed and approved by animal committee of the National Health Research Institutes (NHRI, animal protocol no.: NHRI-IACUC-107098-A and NHRI-IACUC-109088-A). To increase the translation efficiency of CreER^T2^, we used the native internal ribosome entry site (IRES) from encephalomyocarditis virus (EMCV) with A6 bifurcation sequence and an internal initiation codon to generate the CreER^T2^ [25]. The F1A-CreER^T2^ transgenic mice did not show any noticeable phenotype, including fertility and viability. The F1A-CreER^T2^ transgenic mice will be available to the research community.

### 2.2. Crossing of F1A-CreER^T2^ and ROSA26 Mice for Tracing the Activity of F1A-Promoter by LacZ Staining Assay 

The adult hemizygous F1A-CreER^T2^ mice were crossed to homozygous ROSA26, the LacZ reporter mice [26]. The genotypes of F1 mice were determined by PCR with primer pairs: F1A(m)F/Rfp-R and R26F2/R1295 as shown in Supplementary Table S1. The double-positive F1 mice were chosen for the subsequent experiments.

### 2.3. One-Step PCR Genotyping, Genomic DNA Extraction and Polymerase Chain Reaction

Toes (0.1~0.2 cm) from 10-day old mice were treated with 100 μL DirectPCR Lysis Reagent (Viagen Biotech, Los Angeles, CA USA) containing 0.2 mg/mL proteinase K (Roche, Pleasanton, CA, USA) at 55 °C overnight, and the crude lysates were then incubated at 85 °C for 45 min to inactivate proteinase K activity. Centrifuging for 60 s and use 1 µL (after 1:3 dilution with distilled water) of lysate for 20 μL PCR reaction. PCR analysis was performed using KAPATaq ReadyMix DNA polymerase (KK1024, Kapa Biosystems, Wilmington, MA, USA) or KAPATaq LongRange HotStart Ready Mix Reagent (KK3601, Kapa Biosystems). Primers used in genotyping PCR of F1A-CreER^T2^ transgenic mice are listed in Supplementary Table S1: PCR was performed in the ABI7900 PCR System. PCR conditions were 94 °C for 10 min; 35 cycles of 94 °C for 40 s, 55 °C for 30 s, 72 °C for 1 min; and a final extension of 72 °C for 7 min. For primer pairs F1AF/CreR, PCR conditions were denaturation for 3 min at 95 °C; followed by 35 cycles of denaturation for 15 s at 94 °C, 15 s annealing at 55 °C, and 10 min extension at 68 °C; and a final cycle of 68 °C for 7 min. All primers and conditions for PCR and QPCR are listed in Supplementary Tables S1 and S2.

### 2.4. RNA Preparation, Reverse Transcription and Quantitative Polymerase Chain Reaction

Total RNA was isolated from tissues by standard method with TRIzol^®^ reagent (Thermo Fisher Scientific, Waltham, MA USA) and the protocol of RNA isolation kit (Macherey-Nagel, Bethlehem, PA, USA). Three μg of total RNA were used for reverse transcription using RevertAid First strain cDNA Synthesis kit (Thermo Fisher Scientific) with oligo (dT)_18_ as primer in a final 20 μL volume. Quantitative PCR was performed in the ABI7500 Real-Time PCR System using SYBR Fast Universal qPCR master Mix (Kapa Biosystems). Primers used in qPCR analysis are: F1A-exon1: forward: 5′-cccaaagccaagaagccacc-3′; reverse: 5′-tgtgctggtcgctcctgtccct-3′; [9] Cre: forward: 5′-gatttcgaccaggttcgttc-3′; reversed: 5′-gctaaccagcgttttcgttc-3′. Gene expression detection and data analysis were performed using ABI7500 1.41 version software (Thermo Fisher Scientific). The relative quantification of mRNAs was determined by 2^−^^ΔΔCt^ with logarithm transformation. Data are presented as means ± SD.

### 2.5. Animal Perfusion and Tissue Process

Eight-week old F1A-CreER^T2^ mice and F1A-CreER^T2^ x ROSA26 F1 mice were anesthetized by isoflurane (Halocarbon Laboratories, River Edge, NJ, USA) and perfused intracardially with ice-cold phosphate buffered saline (PBS) using peristaltic pump (MP-1000, EYELA, Tokyo, Japan). Tissues were dissected for total RNA extraction or frozen section embedding. To identify the expression pattern of RFP via fluorescence per se in F1A-CreER^T2^ mice, the F1A-CreER^T2^ mice were anesthetized by isoflurane and perfused intracardially with ice-cold PBS without calcium and magnesium (Hyclone). Heart tissues were harvested and fixed in pre-chilled 95% ethanol for 20–24 h at 4 °C, then dehydrated in 4 changes of pre-chilled 100% ethanol for 1 h at 4 °C (Nakagawa et al., 2015), followed by embedding with optimal cutting temperature compound (Sakura Finetek U.S.A., Torrance, CA, USA) and kept in −70 °C. The heart tissues were cryosectioned into 6-μm thickness, rinsed with water, and observed directly under a fluorescent microscope without stained with anti-RFP antibody.

### 2.6. LacZ Staining and Immunohistochemistry

Tissues from mice after intracardial perfusion with ice-cold PBS were fixed in 4% paraformaldehyde (Electron Microscopy Science, Hatfield, PA, USA) for 30 min, equilibrated in 10% (*w*/*w*) sucrose for 3 h, then in 30% (*w*/*w*) sucrose overnight, and frozen in Optimal Cutting Temperature compound (VWR) at −80 °C before cryosectioning. Ten-μm cryosections were used for X-gal (5-bromo-4-chloro-3-indolyl β-D-galactosidase) staining and immunohistochemistry staining. For X-gal staining, slides were post-fixed with 0.2% paraformaldehyde on ice for 10 min, rinsed in PBS with 2 mM MgCl_2_ for 10 min twice, in rinse solution for 10 min and then in X-gal solution at 37 °C overnight [27]. The X-gal stained tissue sections were then immunostained with anti-vimentin (1:1000, ARG66199, Arigo Biolaboratories, Wuhan, China), anti-FGF1 (1:70, sc-7910, Santa Cruz Biotechnolgy, Santa Cruz, CA, USA) and anti-cardiac troponin T (1:500, NBP-67594, Novus, St Charles, MO, USA) antibodies. The detection system was used with Leica kit DS9800. For immunofluorescence staining, tissues were fixed with 4% PFA at 4 °C overnight, then fixed with methanol for 15 min at −20 °C. Blocking tissues were done with 10% donkey serum for 45 min at room temperature. Tissues were stained with anti-vimentin (1:1000, ARG66199, Arigo), anti-FGF1 (1:270, #15, IMC-Lab, Zhunan, Taiwan), and anti-cTnT (1:500, NBP-67594, Novus) antibodies. Tissues were incubated with Alexa 488-conjugated donkey anti-rabbit IgG (1:500, Thermo Fisher) for 2 h at room temperature. Hoechst 33258 (200 ng/mL) was used as nuclear counterstain. Tissue slides were mounted with cover-slips and were inspected using BX51 (Olympus, Miami, FL, USA) microscope.

### 2.7. Tamoxifen Preparation and Administration

Corn oil (C8267, Sigma, St. Louis, MO, USA) was sterilized using 0.2 μm syringe filter (Sartorius, Göttingen, Germany). Tamoxifen (T5648, Sigma, St. Louis, MO, USA) was dissolved in sterilized corn oil at 55 °C for 1 h to make solution of 10 mg/mL or 20 mg/mL and stored at −20 °C, protected from light. The tamoxifen solution was thawed at room temperature and mixed well before use. Eight-week old mice were injected intraperitoneally with 100 μL of 10 or 20 mg/mL tamoxifen solution or corn oil vehicle for five consecutive days. After resting for two days, the mice were injected with tamoxifen again for the next five consecutive days. Mice were sacrificed on the third day after the last injection and analyzed.

### 2.8. Statistical Analysis

Student’s *t*-test was used for comparing two groups. Data were expressed as mean ± standard deviation (SD). Statistical significance was accepted when *p* < 0.05. Statistical differences are indicated by * *p* < 0.05, ** *p* < 0.01 and *** *p* < 0.001. Data and graphics were processed by Excel.

## 3. Results

### 3.1. Expression Levels of CreER^T2^ mRNA of F1A-CreER^T2^ Mice Showed Similar Pattern with Endogenous Fgf1A mRNA

To understand the activation of F1A promoter in vivo, we generated F1A-CreER^T2^ transgenic mice. The transgene was constructed using mouse BAC clone RP23-193N23 including *Fgf1A* 5′-UTR (127.7 kb), and exon 1A sequence as the transgene backbone [10], followed by 714 bp of RFP gene sequences, 536 bp of IRES gene sequences [28], 2001 bp of CreER^T2^ gene sequence and 464 bp of 2X BGH pA gene sequence (Figure 1A and Supplementary Figure S1). We got seventeen founders from four foster mothers, and their genomic DNA were extracted for PCR genotyping. Three founders were confirmed to carry the transgene by designing primers for genotyping of three founder mice #13A, #22A and #25A (Figure 1B). The PCR product by using primer pairs: F1AF/RfpR is 537 bp; F1AF/CreR is 2,217 bp; CreF/CreR is 562 bp (Figure 1B). We checked the expression of Cre by quantitative PCR in these three independent transgenic mouse strains. In the heart tissues, #13A strain mice possess the highest gene expression levels, and the differences of Ct value between #25A and #13A strains and between #22A and #13A strains were 9.3 and 8.5, respectively. In addition, we further check the mouse line #25A/R26R and #22A/R26R, the LacZ signals were all negative in the brain, heart, kidney and gWAT, muscle tissues in these two mouse lines. Therefore, founder #13A was chosen for all the following studies.

**Figure 2:**
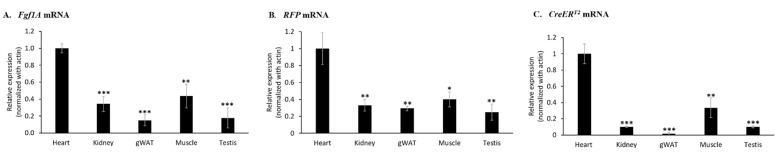
Endogenous *Fgf1A* *RFP* and *CreER^T2^* mRNA expression of F1A-CreER^T2^ transgenic mice. Total RNA from tissues of F1A-CreER^T2^ (#13A) adult male transgenic mice were extracted. (**A**) The levels of *Fgf1A* mRNA were higher in heart, kidney and muscle than in other tissues. Similar expression mRNA patterns could be observed in the (**B**) *RFP* and (**C**) *CreER^T2^* gene, which is driven by the mouse *Fgf1A* promoter. The mRNA expression levels of *Fgf1A, CreER^T2^* and *RFP* were analyzed by quantitative PCR, normalized to β-actin, and compared with the heart tissue. These results are shown as means ± SD, *n* = 3, * *p* < 0.05, ** *p* < 0.01 and *** *p* < 0.001.

In order to investigate whether the mRNA expression levels of endogenous *Fgf1A* gene is correlated with expression of *RFP* and *CreER^T2^* gene, we analyzed the mRNA expression levels in different organs of F1A-CreER^T2^ transgenic mice (#13A), including heart, kidney, gWAT (gonadal white adipose tissue), muscle and testis. Notably, we found that the heart tissue possessed the highest mRNA expression levels of *Fgf1A* (Figure 2A), *RFP* (Figure 2B) and *CreER^T2^* (Figure 2C) genes. In addition, muscle and kidney tissues possessed low mRNA expression levels of *Fgf1A* gene.

### 3.2. Tamoxifen-Activated Cre-loxP Recombination of Genomic DNA Is Tissue-Specific and Dose-Dependent

**Figure 3:**
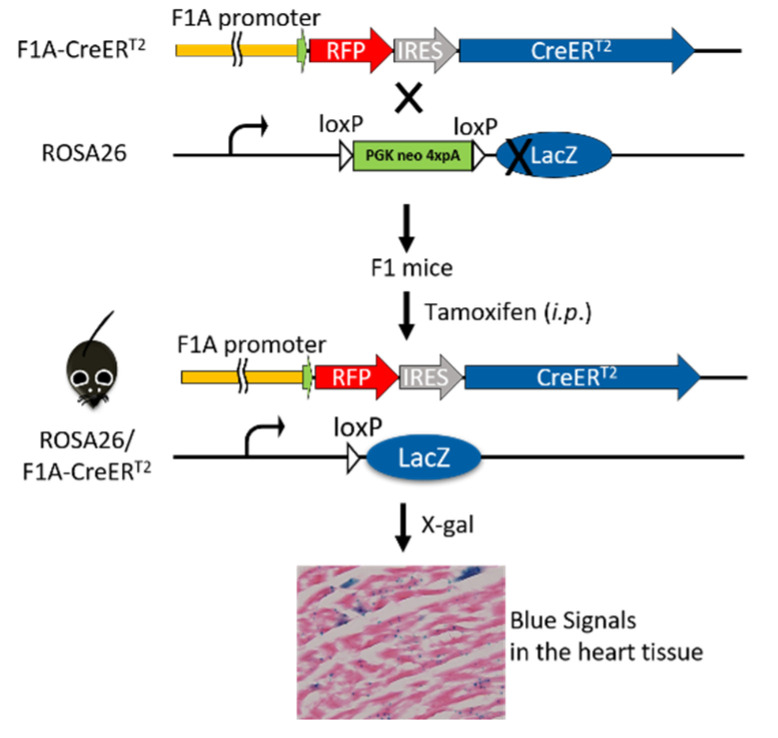
Breeding of F1A-CreER^T2^ × ROSA26 F1 mice. The F1A-CreER^T2^ transgenic mice were crossed to LacZ reporter mouse line, ROSA26, and the double-positive F1 mice were chosen for protein expression analysis. After tamoxifen treatment, the CreER^T2^ recombinase-estrogen receptor fusion protein, which is driven by tissue-specific promoter, F1A, will bind to tamoxifen and translocate into nucleus to recombine its loxP-flanked DNA targets, remove the transcriptional termination poly A cassette and produce β-galactosidase. The activity of β-galactosidase could be detected by staining tissues with X-Gal and showing punctate blue signals.

**Figure 4:**
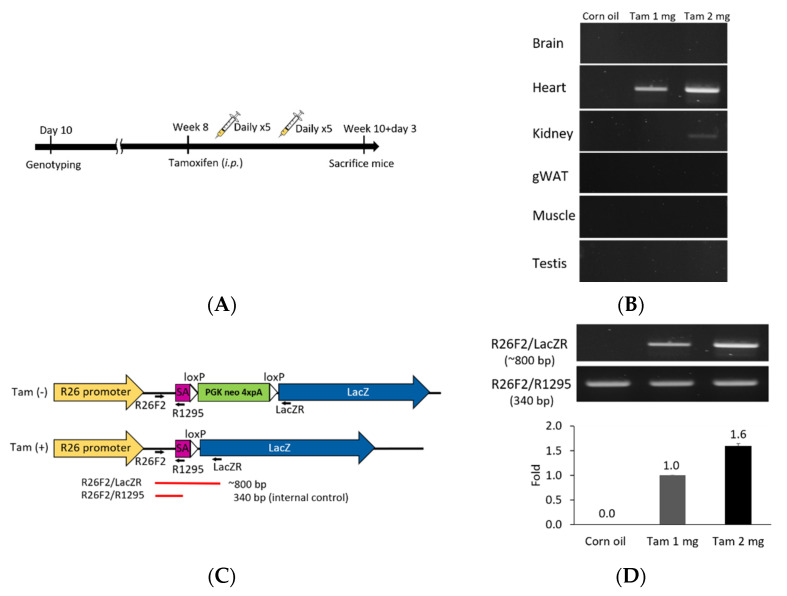
Tamoxifen induction *Cre-loxP* recombination of F1A-CreER^T2^ × ROSA26 F1 mice. (**A**) Eight-week old double-positive F1 mice were injected intraperitoneally with tamoxifen or corn oil vehicle control for 10 days within two weeks. The schematic flow is shown in (**A**). Tamoxifen was injected in eight-week old mice intraperitoneally with 100 μL/mouse/day at 1 or 2 mg per mouse, during five consecutive days in a week, for a total of two weeks. (**B**) Tamoxifen-activated *Cre-loxP* recombination of genomic DNA PCR analysis of gene recombination. (**C**) The primers used for detection of *Cre-loxP* recombination. The purple box represents the splicing acceptor site. The white box represents the *loxP* sequence. (**D**) PCR results for *Cre-loxP* recombination of heart tissues. Tam 1 mg: tamoxifen 1 mg per mouse per day, Tam 2 mg: tamoxifen 2 mg per mouse per day.

We further explored this unique *Fgf1A* and *CreER^T2^* heart-specific gene expression patterns and performed lineage tracing in F1A-CreER^T2^ × ROSA26 mice. Cre/loxP-mediated recombination in tracing cells is irreversible, and the descendants can be identified by their *LacZ* reporter expression (Figure 3). We then utilized the inducible *Cre* allele for ‘pulse-chase’ experiments to trace the *Fgf1A*-expressing cells in the adult heart tissue. CreER^T2^ can translocate into nucleus for recombination of adjacent *loxP* sites, resulting in the deletion of the intercalating transcriptional termination polyA sequence. The CreER^T2^ translocation depends on the addition of tamoxifen at the designated time-points (pulse), which allows for targeting and tracing of lineage cells (chase). In the present study, we generated the F1A-CreER^T2^ line and crossed it with ROSA26 mice. The eight-week-old F1 mice were injected intraperitoneally with tamoxifen or corn oil vehicle control for five consecutive days, then another five consecutive days after two days resting period. Two days later, we then collected tissues from ten-week-old mice (Figure 4A). In order to confirm the tamoxifen-induced Cre-loxP recombination, the PCR analyses for genomic DNA of gene recombination showed the recombination occurred abundantly in the heart, and, to a much lesser extent, in the kidney (Figure 4B). Specific primers used to detect the genomic DNA recombination are according to previous report [27] (Figure 4C), and we found the recombination in the heart was specific. PCR results of R26F2/LacZR, R26F2/R1295, R26F2/LacZR, R26F2/R1295, R26F2/LacZR and R26F2/R1295 are shown in Supplementary Figure S2. Further, the recombination is dose-dependent, as 2 mg per mouse of tamoxifen induced higher levels of *loxP* recombination than 1 mg per mouse of tamoxifen (Figure 4D).

### 3.3. F1A-CreER^T2^ Mice Could Be Used to Trace and Label the Specific Cell Type Activated in the Heart after Tamoxifen Treatment

**Figure 5:**
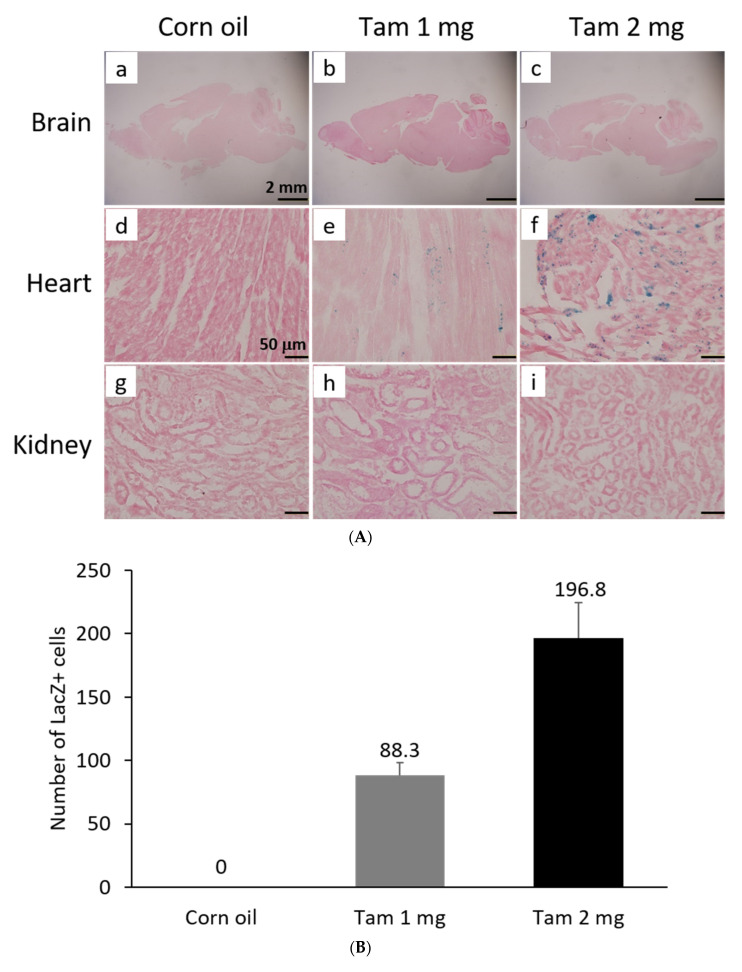

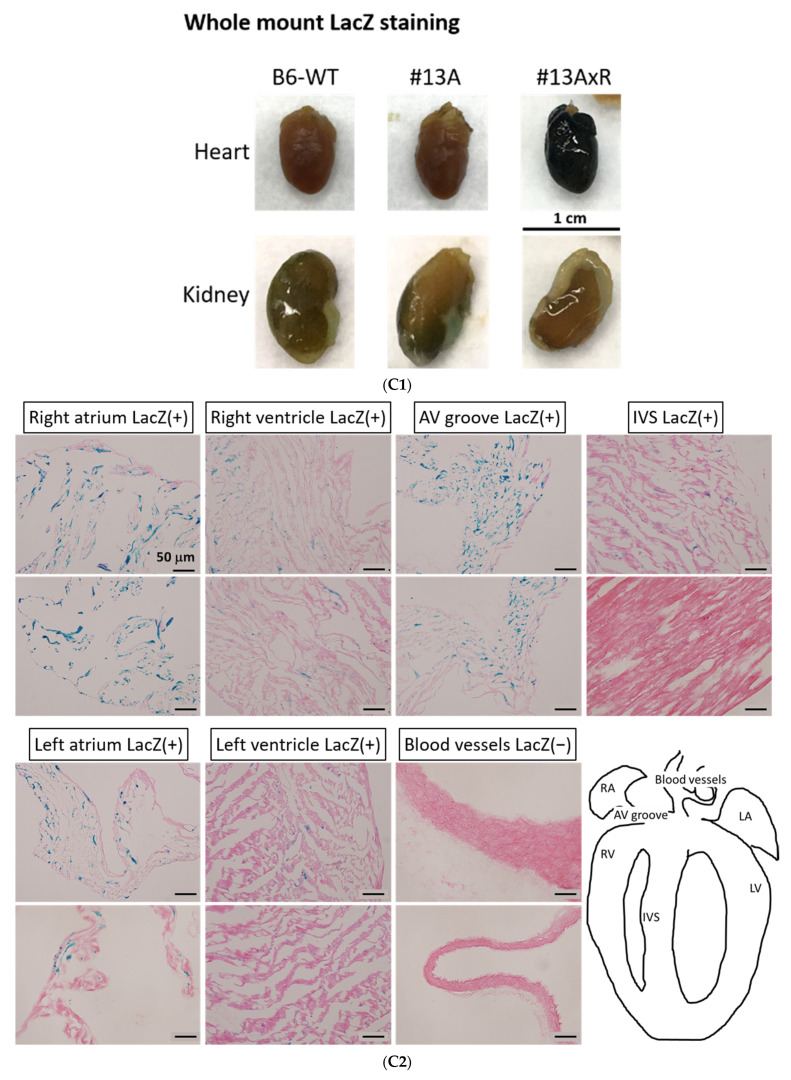

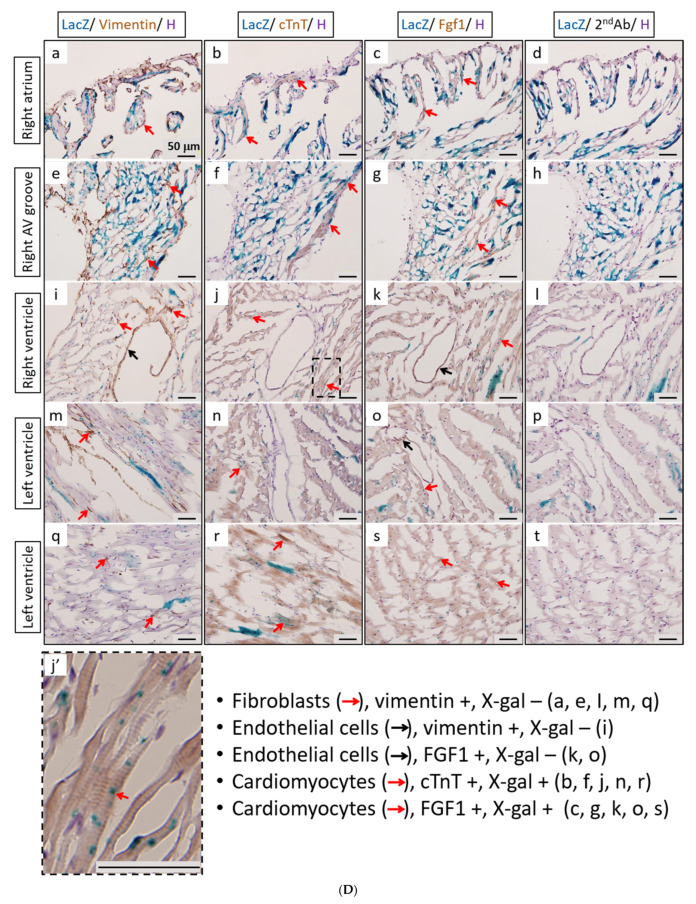
Expression profile of *F1A*-driven expression of *LacZ* gene in F1A-CreER^T2^ x ROSA26 F1 mice. (**A**) LacZ-positive signals showed tissue-specific and dose-dependent manner after tamoxifen treatment. LacZ-positive blue signals of LacZ staining were detected only in heart tissues of F1A-CreER^T2^ x ROSA26 F1 transgenic mice after tamoxifen treatment (Panels **e**,**f**) but not in brain (Panels **b**,**c**) and kidney (Panels **h**,**i**). No LacZ staining could be detected in the absence of tamoxifen administration (Panels **a**,**d**,**g**). Scale bar, 2 mm (**a**–**c**); 50 µm (**d**–**i**). (**B**) The LacZ-positive cells in the heart were counted and the data were expressed as mean ± SEM, *n* = 3. (**C1**) Whole mount LacZ staining of heart and kidney organs. (**C2**) Spatial distribution of LacZ-positive cells in the heart. LA: left atrium, LV: left ventricle, IVS: inter-ventricular septum, RA: right atrium, RV: right ventricle. AV groove: atrium and ventricle groove Scale bar, 50 µm. (**D**) LacZ-positive cells were not co-localized with vimentin-positive fibroblasts and endothelial cells ((**a**,**e**,**i**,**m**,**q**), red arrows). LacZ-positive cells were exclusively co-localized with cTNT-positive cells ((**b**,**f**,**j**,**n**,**r**), red arrows). LacZ-positive cardiomyocytes were co-localized with FGF1-positive cells ((**c**,**g**,**k**,**o**,**s**), red arrows). Of note, some FGF1-positive cells were not only co-localized with cardiomyocyte, but co-localized with endothelial cells ((**k,o**), black arrows), indicating different *Fgf1* promoters were used for different cell types in the heart. (**j’**) A high magnification picture of a portion of Panel (**j**) is to demonstrate cardiac-specificity of the Cre expression. Scale bar, 50 µm. H: Hematoxylin.

The activity of β-galactosidase could be detected by staining tissues with X-Gal and showed many punctate blue signals. Notably, we found that the LacZ-positive blue signals were specifically and dose-dependently induced by tamoxifen at 1 and 2 mg per mouse in the heart, but not in other tissues, such as brain and kidney (Figure 5A). After quantification, our data suggested that (1 mg) and (2 mg) tamoxifen treatment could dose-dependently increase the number of LacZ-positive cells to 88.3 ± 9.8 and 196.8 ± 27.6, respectively (Figure 5B). Moreover, we utilized whole heart staining with X-Gal to demonstrate that LacZ-positive blue signals were only expressed in the ROSA26/F1A-CreER^T2^ mice (Figure 5C1). Using heart tissue sections of ROSA26/F1A-CreER^T2^ mice for the staining of X-Gal, we further demonstrated that LacZ-positive signals were detected in the right atrium (RA), right ventricle (RV), atrium and ventricle (AV) groove, IVS, left atrium (LA), left ventricle (LV) of the heart (Figure 5C2). Notably, the LacZ-positive signals were not observed in blood vessels (Figure 5C2). In order to investigate whether any specific patterns in a spatial distribution of LacZ-positive cardiomyocytes, in terms of LA, RA, LV, RV, IVS and blood vessels, we also provided the data of spatial distribution of LacZ-positive cells in other F1A-CreER^T2^(#13A)/ROSA26 mice, including animals #15, #16, #29, #30, #36, #39, #42, #54 (Table 1). For all the heart tissues from tamoxifen-treated F1A-CreER^T2^(#13A)/ROSA26 mice, we invariably detected the LacZ staining as shown in Table 1. To further characterize the LacZ-positive cells, we utilized anti-vimentin antibody to detect fibroblasts and endothelial cells and anti-cardiac troponin T (cTNT) antibody to stain cardiomyocytes. We found that LacZ-positive cells were not co-localized with vimentin-positive fibroblasts and endothelial cells (Figure 5D(a,e,i,m,q), red arrows). We further found that co-localization of LacZ-positive cells and cTNT-positive cells were observed in these tissues (Figure 5D(b,f,j,n,r), red arrow) indicating the unique and cardiomyocyte-specific pattern of *Fgf1A* mRNA expression in the heart. We further used anti-FGF1antibody to demonstrate all of the LacZ-positive cardiomyocytes were co-localized with FGF1-positive cells (Figure 5D(c,g,k,o,s), red arrows). Of note, some FGF1-positive cells were not co-localized with cardiomyocytes, but co-localized with endothelial cells (Figure 5D(k,o), black arrows), suggesting that different *Fgf1* promoters might be activated for different cell types in the heart. We further provided a high magnification picture (Figure 5D(j’)) to demonstrate cardiac-specificity of the Cre expression.

### 3.4. The Specific Cell Types Labeled in the Heart with F1A Promoter-Driven RFP Expression in F1A-CreER^T2^ Mice

**Figure 6:**
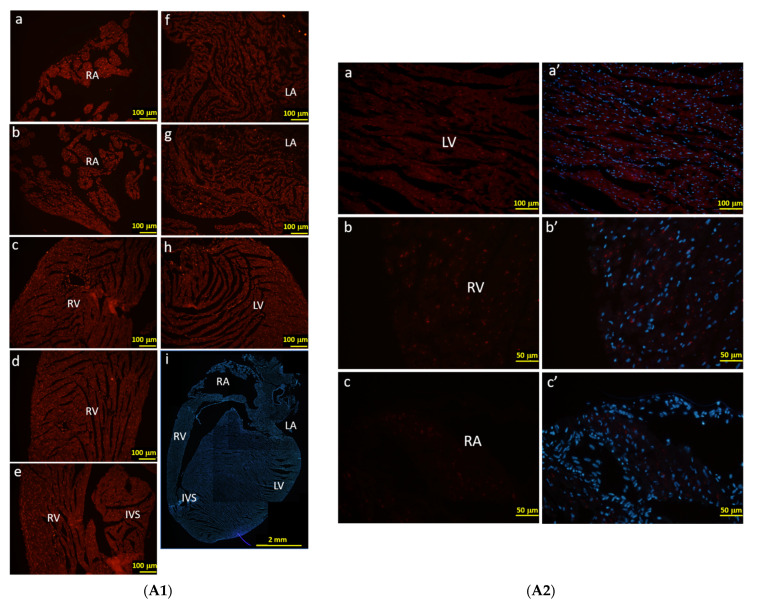

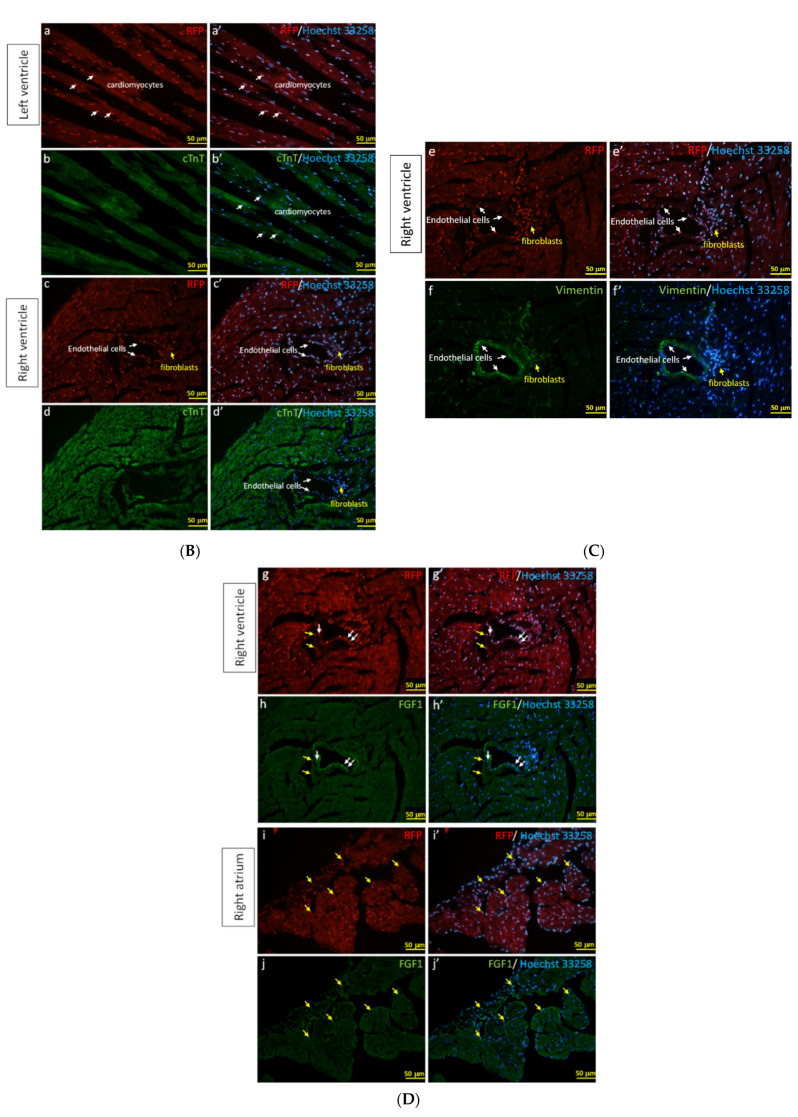
The RFP expression patterns of F1A-CreER^T2^ transgenic mice correlate with FGF1 IHC staining. (**A1**) RFP-positive signals were detected in heart tissues of F1A-CreER^T2^ transgenic mice. Spatial distribution of RFP-positive cells in the heart (**i**). LA: left atrium (**f**,**g**), LV: left ventricle (**h**), IVS: inter-ventricular septum (**e**), RA: right atrium (**a**,**b**), RV: right ventricle (**c**,**d**). AV groove: atrium and ventricle groove. Scale bar, 100 µm (**a**–**h**); 2 mm (**i**). (**A2**) RFP (**a**–**c**) and Hoechst signals (**a’**–**c’**) in wild type mice. Scale bar, 100 µm (**a**,**a’**); 50 µm (**b**–**c’**). (**B**) RFP-positive cells showed correlated distribution with cTNT-positive cardiomyocytes in the left (**a**–**b’**) and right ventricles (**c**–**d’**). (**C**) RFP-positive cells showed correlated distribution with vimentin-positive endothelial cells and fibroblasts in the right ventricles (**e**–**f’**). (**D**) RFP-positive cells showed correlated distribution with FGF1-positive endothelial cells, fibroblasts and cardiomyocytes in the right ventricles (**g**–**j’**). Scale bar, 50 µm.

In order to characterize the RFP-positive cells that were activated by F1A promoter, we observe the RFP signals directly under the fluorescence microscope without immunohistochemical staining using anti-RFP antibody. Consistently, we could observe the RFP-positive cells in the regions of RA, RV, AV, IVS, LA and LV of heart (Figure 6A), which were similar to our observations of LacZ staining in Figure 5C2. We further demonstrated the RFP-positive cells were co-localized with cTnT-positive cardiomyocytes and FGF1-positive cardiomyocytes in the RA region (Figure 6B). RFP-positive cells were also co-localized with endothelial cells and fibroblasts in the RV and LV regions (Figure 6C). Consistently, RFP expression in cells could recapitulate the expression of FGF1 in cardiomyocytes in RA region and in fibroblasts and endothelial cells in RV region (Figure 6C,D). In Figure 6A2, the native RFP signal could not be detected in the heart cells of WT mice, neither cytoplasm nor nucleus. These negative results were in stark contrast with the RFP signals detected in the F1A-CreER^T2^ mice (Figure 6A1). In a higher resolution in Figure 6B, RFP expression was distinctly expressed in the nuclei of the cardiomyocytes. It is interesting to note that most of the RFP signals are localized to the nuclei. In addition, we have observed that the levels of native RFP expression were more robust surrounding the atrium and ventricle. Notably, the levels of RFP expression were lower in the central region of the left ventricle in the heart of F1A-CreER^T2^ mice (data not shown). Taken together, F1A promoter-driven RFP expression in the specific cell types in the heart was consistent with LacZ-positive signals in the heart of F1A-CreER^T2^ mice. We further provide the comparison of LacZ-positive cells and RFP-positive cells in different regions of heart tissues in F1A-CreER^T2^ and ROSA26/F1A-CreER^T2^ mice, respectively (Table 2). Immunohistochemical stainging results in the heart of E18 embryo of F1A-CreER^T2^ after tamoxifen treatment are shown in Supplementary Figure S3.

## 4. Discussion

FGF1 is important in regulating many biological and physiological processes. It is of great importance to note that subcutaneously injection of mouse FGF1 or intracerebroventricular injection of human FGF1 can significantly improve the insulin sensitivity and reduce the serum glucose levels in mouse models of diabetes [29]. In myocardial infarction, FGF1 has been shown to regulate angiogenesis [30,31] and induce cardiomyocyte regeneration and improves function recovery of the heart [19]. We previously demonstrated that FGF1 can stimulate cardiogenesis through FGF1/FGFR/PKC signaling axis [20]. FGF1 and FGFR signaling can induce cell cycle reentry in neonatal cardiomyocytes [21]. Furthermore, FGF1 treatment has been shown to induce cardiomyocyte mitosis, improve heart function and increase angiogenesis in rat hearts with myocardium infarction [19].

Mouse *Fgf1* gene comprises three tissue-specific promoters, which generates three alternatively spliced transcripts (*Fgf1A*, *Fgf1B* and *Fgf1G*). We have previously demonstrate that the *Fgf1A* transcript is predominantly expressed in heart and kidney [10]. In the high-fat diet-induced obesity mouse model, abundant *Fgf1A* expression was detected in adipose tissue in wild-type mice. It has been reported that *Fgf1* can regulate the homeostasis of adipose tissues and *Fgf1* knockout mice showed an aggressive diabetic phenotype after feeding with high-fat diet [18]. Remarkably, subcutaneously injection of FGF1 protein can significantly improve the insulin sensitivity and reduce the serum glucose levels in two mouse models of diabetes (ob/ob, db/db and diet-induced obese insulin resistant mice). Significant reduction of serum glucose levels can be achieved within 24 h, and last more than 48 h. In addition, a higher dose of FGF1 did not result in hypoglycemia in the diabetic mice [32]. Pretreatment of FGF1 can also significantly reduce the serum glucose level in streptozotocin-induced diabetic mice with the loss of β cells [32]. Furthermore, long-term FGF1 administration can ameliorate the systemic inflammation in ob/ob mice, acting through FGFR1 pathway and functions like thiazolidinedione as an insulin sensitizer [32]. Furthermore, it was demonstrated that a single intracerebroventricular FGF1 injection can reduce the disease progress in rodent models of type 2 diabetes [29]. Notably, it has been demonstrated that the serum level of FGF1 is significantly elevated in newly diagnosed type 2 diabetes and correlated with metabolic parameters, such as fasting plasma glucose and body mass index [33]. *Fgf1* gene knockout mice further developed diabetes coupled with aberrant adipose enlargement [19].

From our data, the LacZ expression could be detected in cardiomyocytes, but not fibroblasts and endothelial cells, indicating FGF1 may function through autocrine manner on cardiomyocytes, but through paracrine manner on fibroblasts and endothelial cells. It has been shown that FGF1 is crucial for cell cycle reentry of cardiomyocytes through interaction of FGFR-1 and fibroblast growth factor-inducible molecule 14 (Fn14) [21]. Furthermore, Cx43-knockdown significantly increased the proliferation of neonatal cardiomyocytes through upregulation FGF1 expression in an autocrine manner [13]. In the literature, it has been suggested that the stimulatory effects of FGF1 on endothelial cells to promote angiogenesis and proliferation [19,34].

We have demonstrated that the mRNA expression patterns of *Fgf1A*, CreER^T2^ and RFP were correlated in different organs of the F1A-CreER^T2^ transgenic mice, indicating that F1A-CreER^T2^-derived RFP signal could recapitulate the *Fgf1* mRNA expression in the tissues of F1A-CreER^T2^ transgenic mice. In addition, we have observed that the levels of native RFP expression were more robust surrounding the atrium and ventricle. Notably, the levels of RFP expression were lower in the central region of the left ventricle in the heart of F1A-CreER^T2^ mice (data not shown). It would be worthwhile to investigate the role of endogenous *Fgf1* in cardiomyocyte mitosis and cell cycle reentry upon myocardial infarction in the future. Promoter 1A has been shown to be involved in obesity, diabetes, and other metabolic diseases. It is important that we now identify 1A promoter to be activated in cardiac tissues. This line would be useful for researchers to study different metabolic diseases [18] and regulation of cardiogenesis [10,19,20,21]. Therefore, the establishment of genetic tool that can trace *Fgf1A* expression in vivo will be helpful for delineating the physiological role of *Fgf1A* in heart failure, obesity and diabetes.

## 5. Conclusions

F1A-CreER^T2^ line is a valuable tool for in vivo lineage tracing of *Fgf1A*-expressing cells during heart development and will facilitate the elucidation of the physiological and pathological role of *Fgf1A* in heart failure and obesity.

## Figures and Tables

**Table 1:** Summaries of LacZ-positive signals of heart tissues in F1A-CreER^T2^/ROSA26 mice.

Animal No.	LA	LV	IVS	RA	RV	Blood Vessels
#15	/	+	+	/	+	/
#16	+	+	/	/	+	/
#29	/	+	+	+	+	Neg.
#30	/	+	/	/	+	Neg.
#36	/	+	/	/	+	/
#39	+	+	+	/	+	Neg.
#42	+	+	+	+	+	Neg.
#54	+	+	+	+	+	Neg.

LA, left atrium; LV, left ventricle; IVS, inter-ventricular septum; RA, right atrium; RV, right ventricle; Neg., negative; /, tissue section not available.

**Table 2:** Comparison of LacZ-positive cells and RFP-positive cells in different regions of heart tissues in F1A-CreER^T2^/ROSA26 and F1A-CreER^T2^ mice, respectively (A,B).

A				
	RA	AV Groove	RV	LV
LacZ (+)	cTnT(+) cardiomyocytes Vimentin(−) FGF1(+)	cTnT(+) cardiomyocytes Vimentin(−) FGF1(+)	cTnT(+) cardiomyocytes Vimentin(−) FGF1(+)	cTnT(+) cardiomyocytes Vimentin(−) FGF1(+)
RFP (+)	cTnT(+) cardiomyocytes Vimentin(+) fibroblasts FGF1(+)	NA	cTnT(+) cardiomyocytes Vimentin(+) fibroblasts Vimentin(+) endothelial cells FGF1(+)	cTnT(+) cardiomyocytes FGF1(+)
**B**				
**Markers**		**Cardiomyocytes**	**Fibroblasts**	**Endothelial Cells**
LacZ		+	ND	ND
RFP		+	+	+
FGF1		+	+	+

NA: not available. ND: not detectable.

## Data Availability

Not applicable.

## Supplementary Materials

The following are available online at https://www.mdpi.com/article/10.3390/cells11010121/s1, Figure S1: Construction map of the mouse F1A-CreERT2 transgene, Figure S2: PCR results of R26F2/LacZR, R26F2/R1295, R26F2/LacZR, R26F2/R1295, R26F2/LacZR and R26F2/R1295 (internal control). Figure S3: LacZ stainging in the heart of E18 embryo. Supplementary Table S1: Primers for PCR, Supplementary Table S2: Primer for qPCR.
