# Supplementary material for: Establishing F1A-CreERT2 Mice to Trace Fgf1 Expression in Adult Mouse Cardiomyocytes
Source: Cells. 2021 Dec 30;11(1):121. doi: 10.3390/cells11010121 (PMC8749990; doi:10.3390/cells11010121)
Supplement: Supplementary file 1 [file cells-11-00121-s001.zip › cells-1485381-supplementary.pdf]

**Supplementary Table S1.** Primers for PCR and Table2. Primer for qPCR

| Amplicon | Expected size | Primer pair | Primer name | Sequences 5'→3' |
|---|---|---|---|---|
| F1A/RFP | 537 bp | Forward | F1A(m)-F | gccacctgttaagtggccag |
| | | Reverse | Rfp-R | cgtcagcggggtacagcatc |
| Cre | 562 bp | Forward | CreF | ctaaactagcttcatcgtcggtc |
| | | Reverse | CreR | tctgaccagagtcatccttagcg |
| LacZ | 336 bp | Forward | LacZF | actggccgtcgttttacaacg |
| | | Reverse | LacZR | ttctccgtgggaacaaacgg |
| R26R_ wild type | 603 bp | Forward | R26F2 | aaagtcgctctgaggttgttat |
| | | Reverse | R523 | ggagcggagaaatggatatg |
| R26R_ transgene | 340 bp | Forward | R26F2 | aaagtcgctctgaggttgttat |
| | | Reverse | R1295 | gcgaagagtttgtcctcaacc |
| GAPDH | 500 bp | Forward | Gapdh-F3 | aaatggtgaaggtcggtgtga |
| | | Reverse | Gapdh-R3 | tcataacggcggttcattcat |
| PGK neo 4xpA Floxed | ~800 bp | Forward | R26F2 | aaagtcgctctgaggttgttat |
| | | Reverse | LacZR | ttctccgtgggaacaaacgg |

**Supplementary Table S2.** PCR results of R26F2/LacZR, R26F2/R1295, R26F2/LacZR, R26F2/R1295, R26F2/LacZR and R26F2/R1295 (internal control)

| Amplicon | Expected size | Primer pair | Primer name | Sequences 5'→3' |
|---|---|---|---|---|
| F1A/exon1 | 499 bp | Forward | F1A-Fw | cccaaagccaagaagccacc |
| | | Reverse | Exon1-Rv | tgtgctggtcgctcctgtccct |
| Cre | 195 bp | Forward | Cre-Fw | gatttcgaccaggttcgttc |
| | | Reverse | Cre-Rv | gctaaccagcgttttcgttc |
| RFP | 333 bp | Forward | RFP-Fw | tgagaatcaaggtggtcgag |
| | | Reverse | RFP-Rv | cgtcagcggggtacagcatc |
| β-actin | 233 bp | Forward | β-actin-Fw | ctaggcaccagggtgtgatg |
| | | Reverse | β-actin-Rv | gttggccttagggttcaggg |

PCR conditions

Kapa LongRange Hotstart Reagent ReadyMix (kk3601)

Amplicon: F1A/Cre (2217 bp) :

PCR program: 1 cycle of 95 ℃ for 5 min, 35 cycles of 95 ℃ for 15 sec, 55 ℃ for 15

sec, 68 ℃ for 10 min and final 68℃ for 10 min。

Kapa Taq ReadyMix with dye (kk1024)

Amplicon: F1A/RFP、Cre、LacZ、RFP : PCR program: 1 cycle of 95 ℃ for 5 min, 35 cycles of 95 ℃ for 30sec, 56 ℃ for 40 sec, 72 ℃ for 1 min and final 72 ℃ for 7 min.

Amplicon: actin、GAPDH: 1 cycle of 95 ℃ for 5 min, 20 cycles of 95 ℃ for 20sec, 55 ℃ for 30 sec, 72 ℃ for 50 sec and final 72 ℃ for 7 min.

qPCR : KAPA SYBR® FAST qPCR Master Mix (2X) Kit

Gene expression detection and data analysis were performed using ABI7500 1.41 version software (ABI). The program is 1cycle of 50 ℃ for 2min, 1 cycle of 95 ℃ for 10min, 40 cycles of 95 ℃ for 15 sec, 60℃ for 1min and 1cycle of 95 ℃ for 15sec 60 ℃ for 1min 95 ℃ for 15 sec 60 ℃ for 15 sec.

**Supplementary Figure S1:**

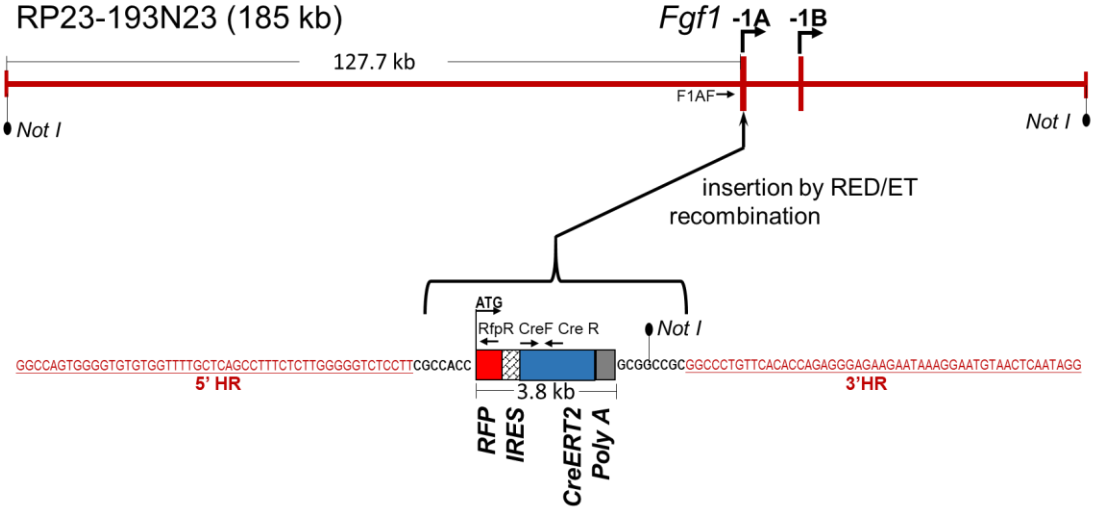


**Supplementary Figure S1:** Construction map of the mouse *F1A-CreER$^{T2}$* transgene. The transgene was constructed using mouse BAC clone RP23-193N23 including *Fgf1A* 5'UTR (127.7 kb), exon 1A, and 1B sequence as the transgene backbone. The bicistronic expression cassette RFP-IRES-CreER$^{T2}$-polyA containing a upstream Kozak sequence and a downstream *Not I* site was inserted into the exon 1A by RED/ET recombination system. The modified BAC was purified, *Not I* digest, and pulsed-field gel electrophoresis to isolate the transgene including the *Fgf1A* 5'UTR and expression cassette for mouse pronuclear microinjection. The junction sequences flanking the insertion site were also shown. Primers designed for genotyping: F1AF, RfpR, CreF and CreR.

**Supplementary Figue S2. PCR results of R26F2/LacZR, R26F2/R1295, R26F2/LacZR, R26F2/R1295, R26F2/LacZR and R26F2/R1295 (internal control)**

Primer pairs: R26F2/LacZR

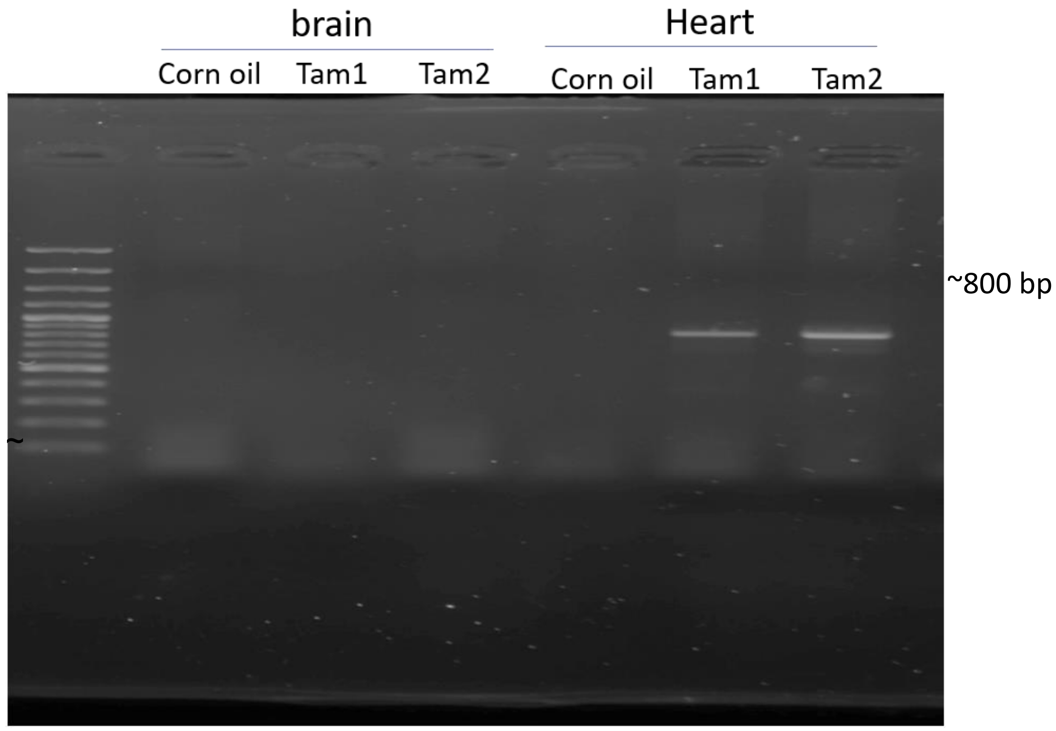


Primer pairs: R26F2/R1295 (internal control)

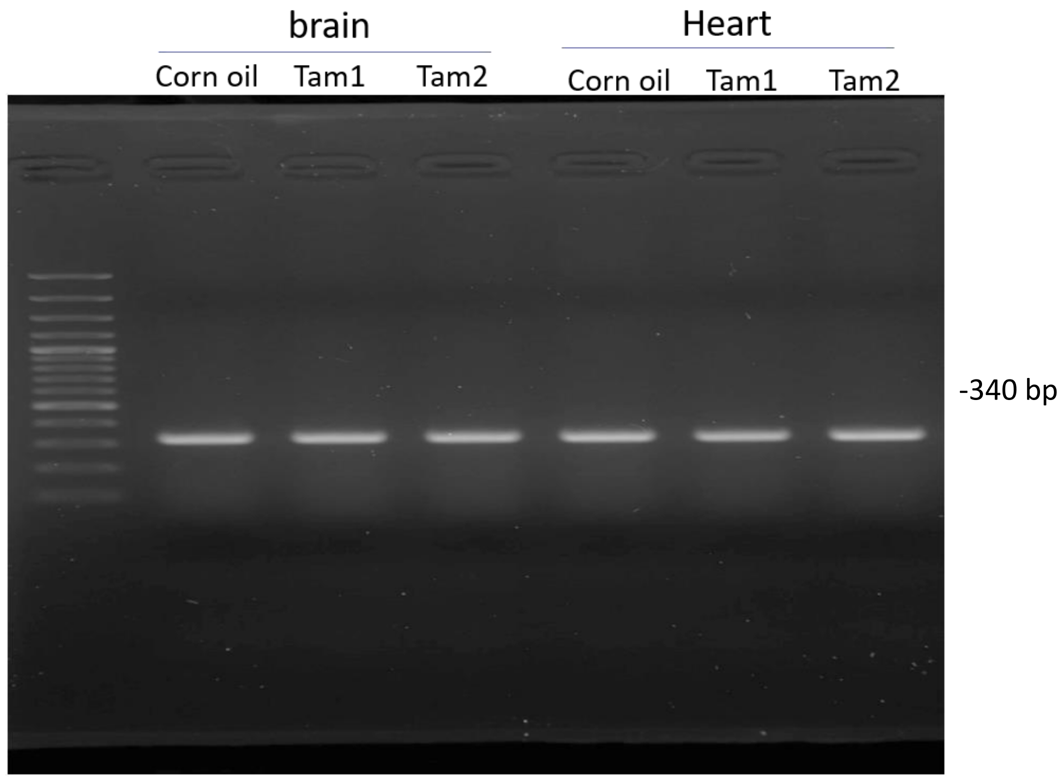

Primer pairs: R26F2/LacZR

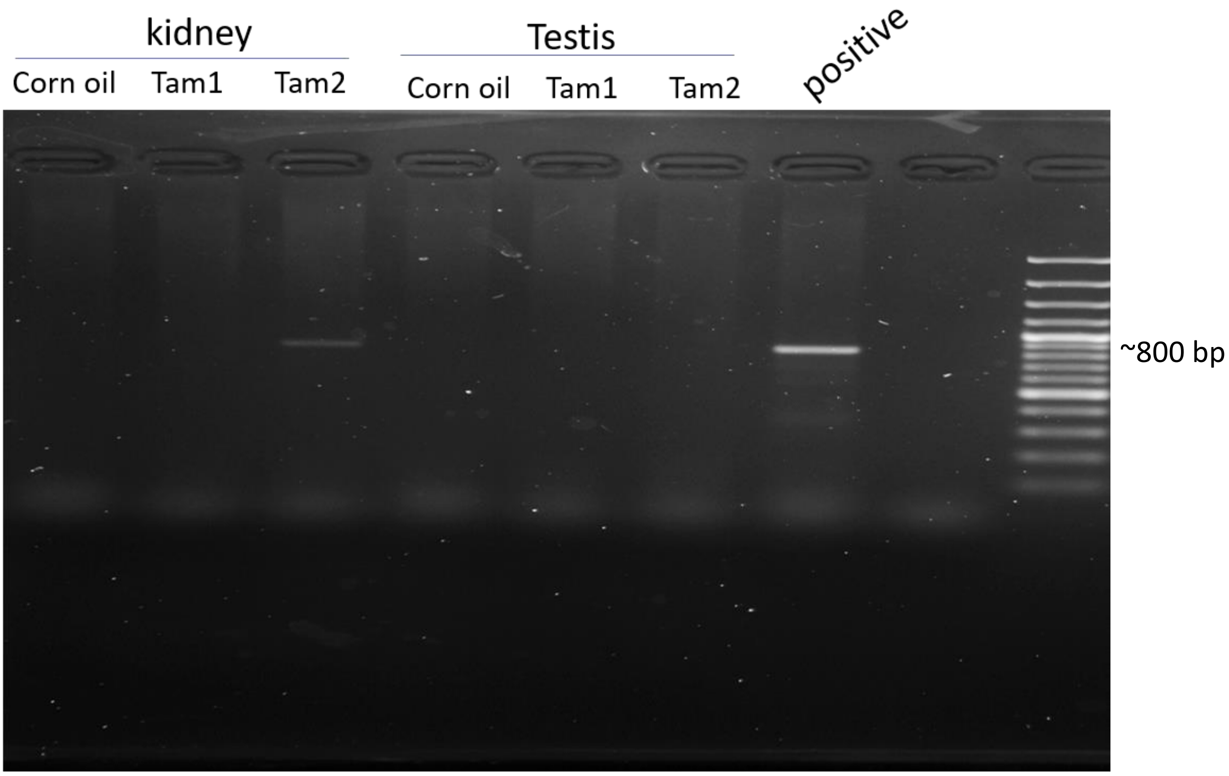


Primer pairs: R26F2/R1295 (internal control)

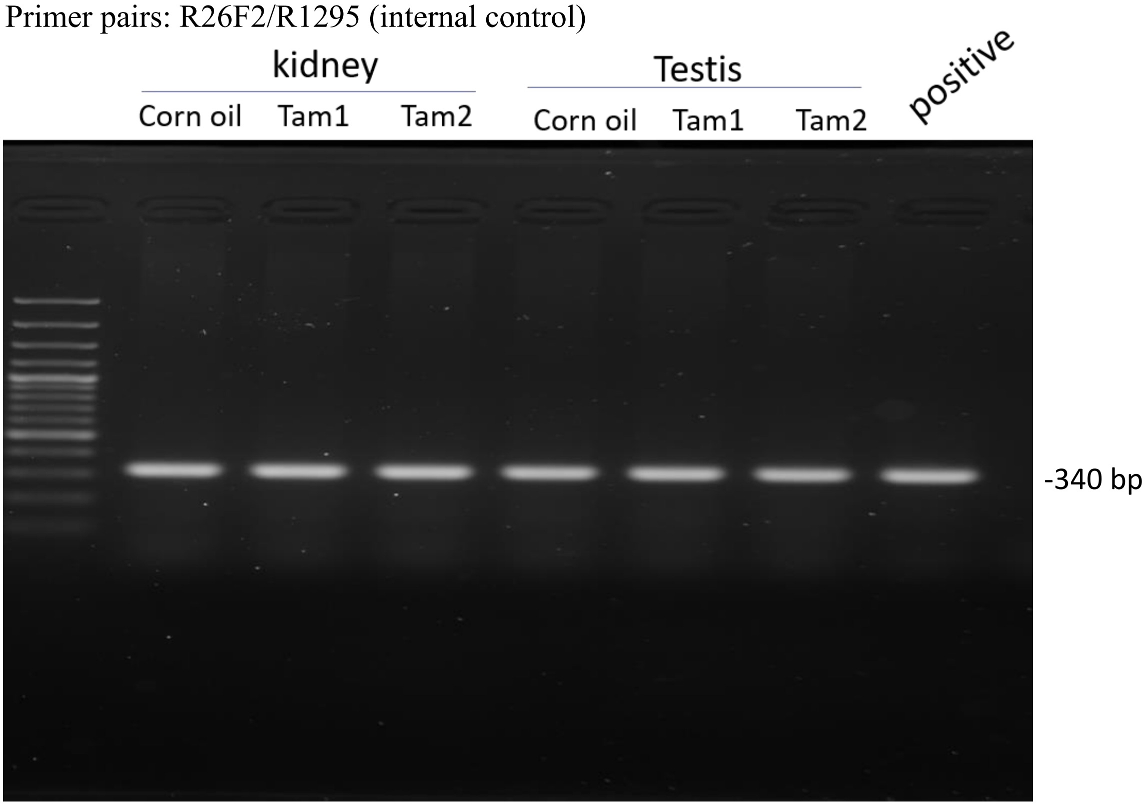

Primer pairs: R26F2/LacZR

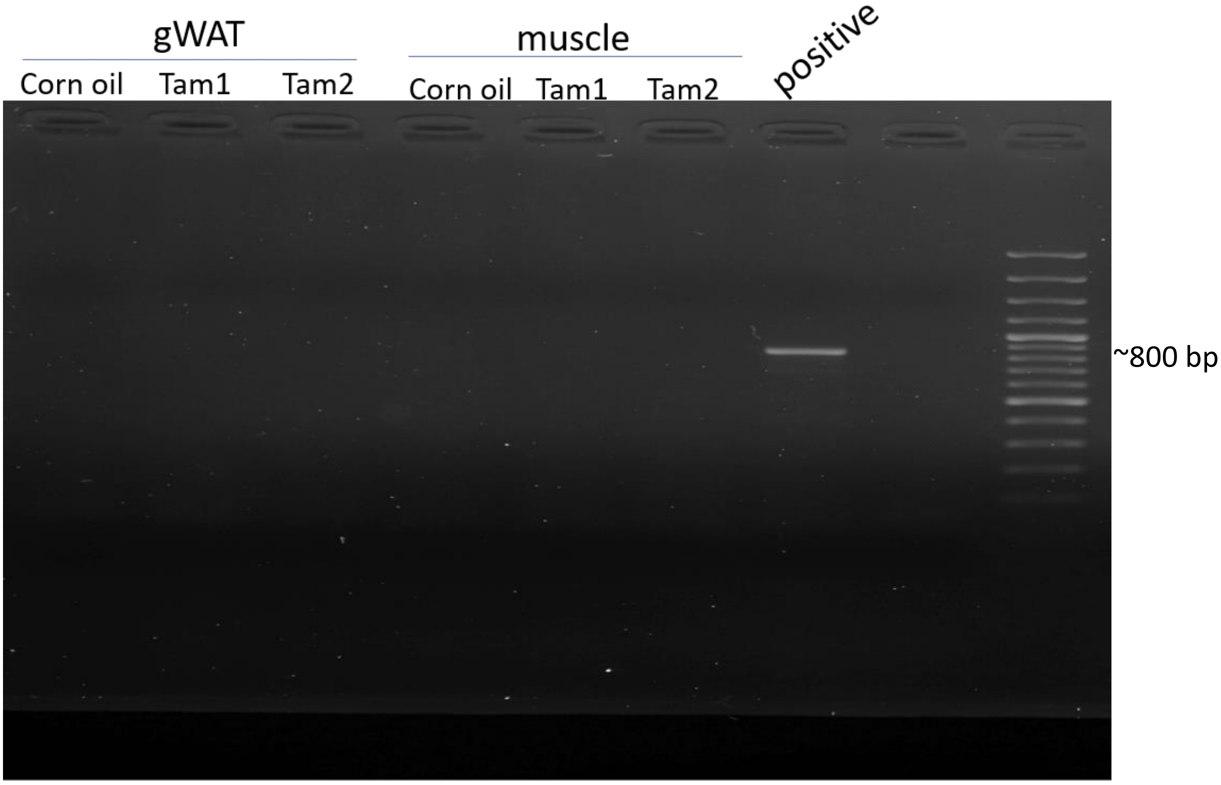


Primer pairs: R26F2/R1295 (internal control)

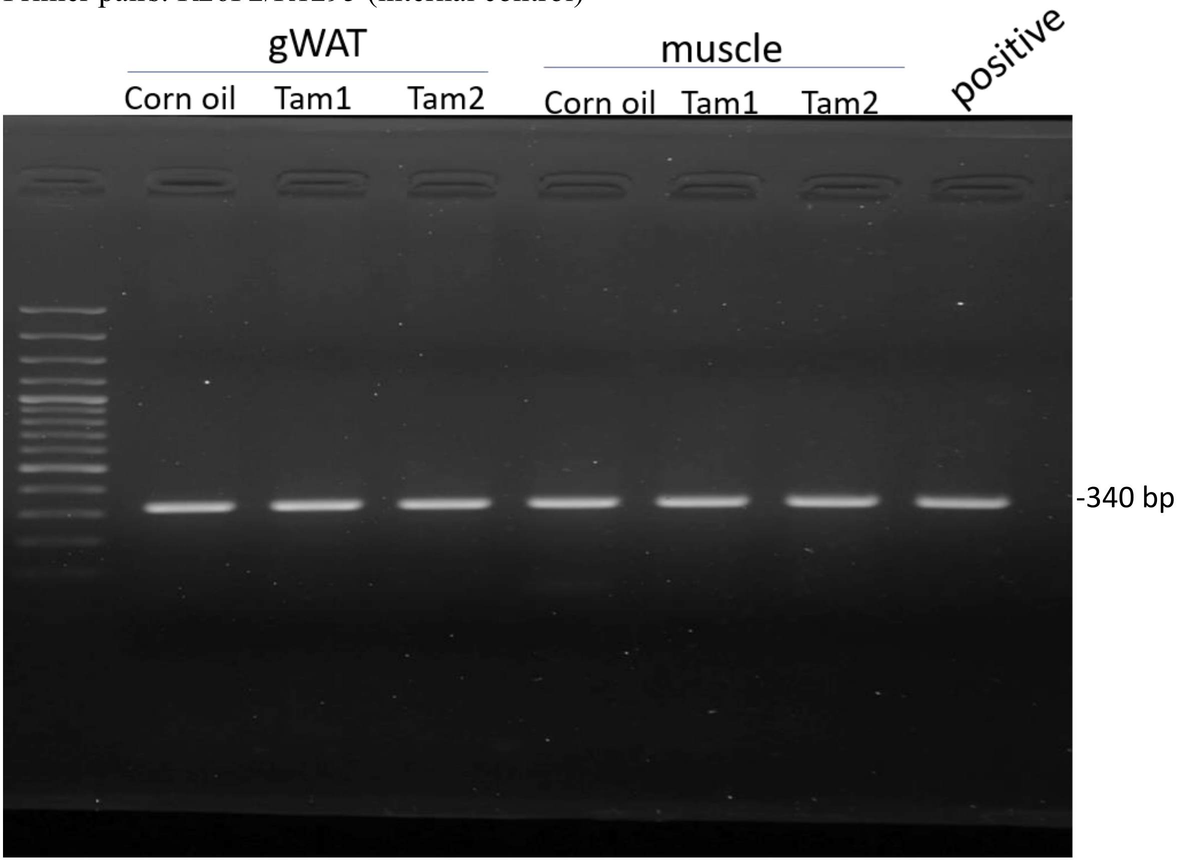

**Supplement Figure S3.** Immunohistochemical stainging in the heart of E18 embryo

A.

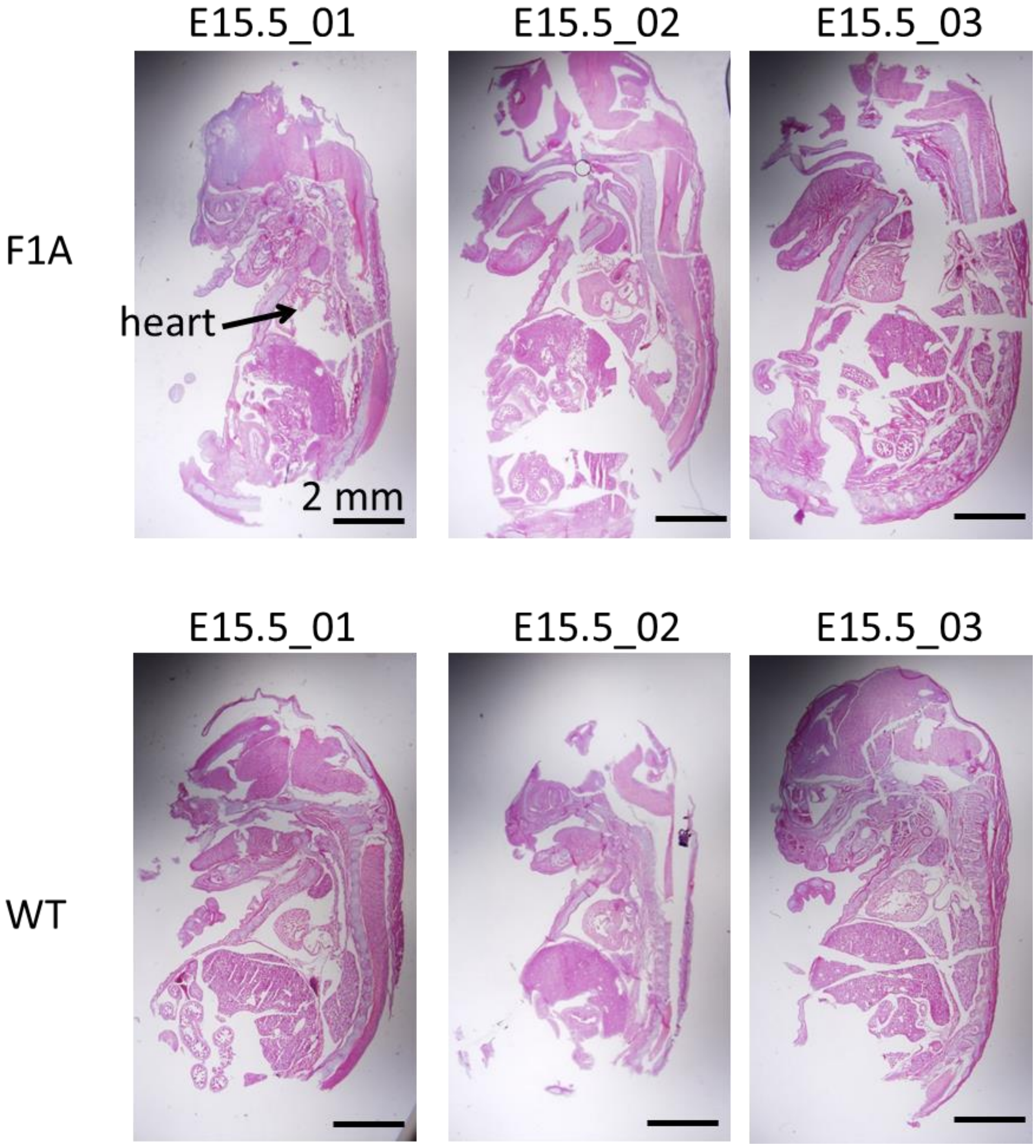

B.

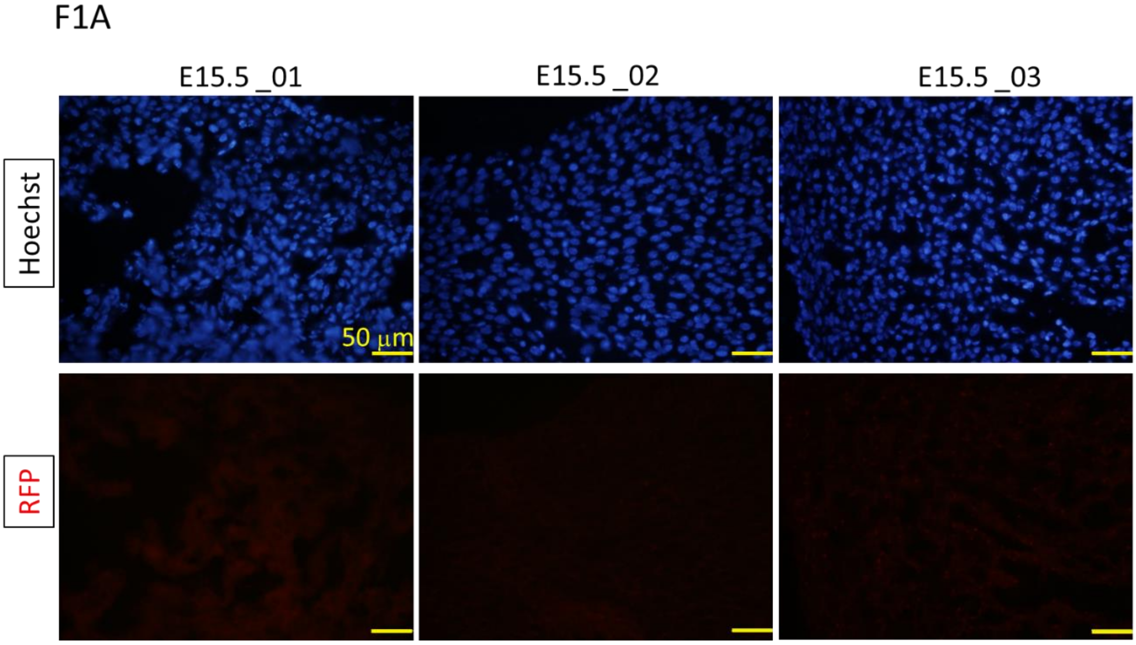
F1A
E15.5 _01
E15.5 _02
E15.5 _03
Hoechst
RFP
50 μm

C.

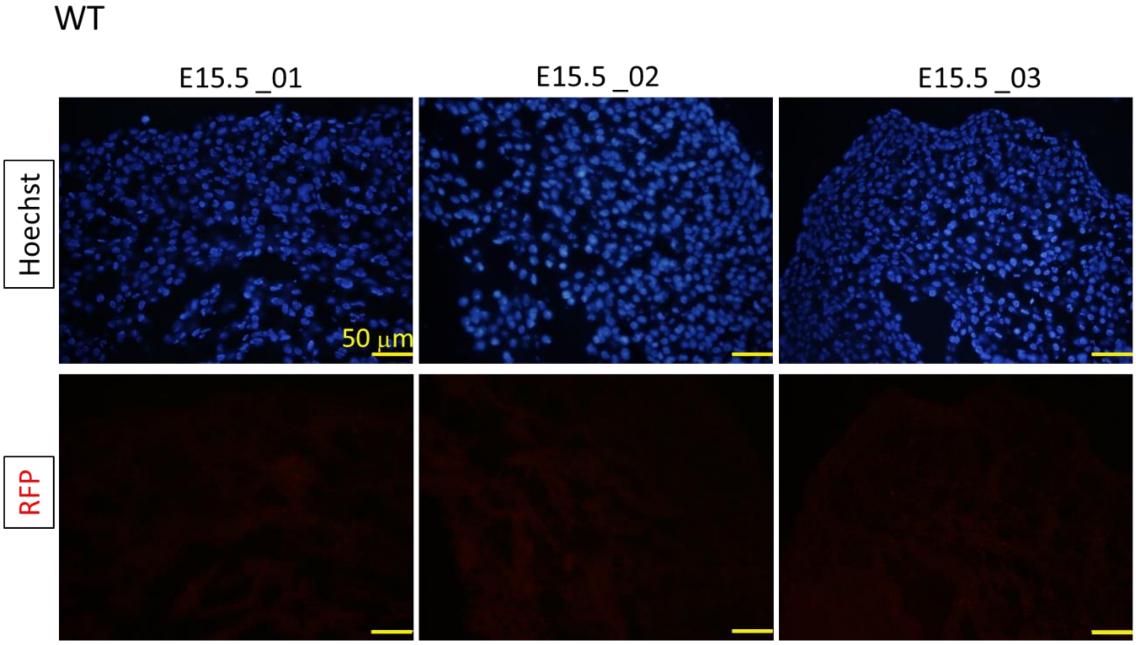
WT
E15.5 _01
E15.5 _02
E15.5 _03
Hoechst
RFP
50 μm
